# 1525. The Impact of Traveler-Based Genomic Surveillance on SARS-CoV-2 Variant Detection in Arriving International Air Travelers– United States, November 29, 2021-April 24, 2022

**DOI:** 10.1093/ofid/ofac492.087

**Published:** 2022-12-15

**Authors:** Grace D Appiah, Teresa C Smith, Renee Wegrzyn, Robert C Morfino, Sarah Guagliardo, Scott Milford, Scott Milford, Allison Taylor Walker, Ezra T Ernst, William W Darrow, Siyao Lisa Li, Thomas Aichele, Andrew Rothstein, Benjamin Rome, Duncan MacCannell, Gabrielle Woronoff, Keith Robison, Dongjuan Dai, Brintha Girinathan, Allison Hicks, Bryan Cosca, Alex Plocik, Birgitte Simen, Leah Moriarty, Martin S Cetron, Cindy R Friedman, Cindy R Friedman, Cindy R Friedman, Cindy R Friedman

**Affiliations:** Centers for Disease Control and Prevention, Atlanta, Georgia; CDC, Atlanta, Georgia; Ginkgo Bioworks Inc, Washington DC, District of Columbia; Ginkgo Bioworks Inc, Washington DC, District of Columbia; CDC, Atlanta, Georgia; XpresSpa Group, Inc., New York, New York; XpresSpa Group, Inc., New York, New York; CDC, Atlanta, Georgia; XpresSpa Group, Inc, New York, New York; XpresSpa Group, Inc., New York, New York; Ginkgo Bioworks, Inc, Boston, Massachusetts; Ginkgo Bioworks, Inc, Boston, Massachusetts; Ginkgo Bioworks, Inc, Boston, Massachusetts; Ginkgo Bioworks, Inc, Boston, Massachusetts; CDC, Atlanta, Georgia; Ginkgo Bioworks, Inc, Boston, Massachusetts; Ginkgo Bioworks, Inc., Boston, Massachusetts; Ginkgo Bioworks, Inc., Boston, Massachusetts; Ginkgo Bioworks, Inc., Boston, Massachusetts; Ginkgo Bioworks, Inc., Boston, Massachusetts; Ginkgo Bioworks, Inc., Boston, Massachusetts; Ginkgo Bioworks, Inc., Boston, Massachusetts; Ginkgo Bioworks, Inc., Boston, Massachusetts; CDC, Atlanta, Georgia; Centers for Disease Control and Prevention, Atlanta, Georgia; CDC, Atlanta, Georgia; CDC, Atlanta, Georgia; CDC, Atlanta, Georgia; CDC, Atlanta, Georgia

## Abstract

**Background:**

International travel facilitates SARS-CoV-2 spread globally. Early detection of variants among arriving international travelers could provide viral information about introduction of variants with differing infectivity, virulence, and vaccine effectiveness, enabling adjustments to treatment and prevention strategies. We initiated a genomic surveillance program at 4 US airports to detect SARS-CoV-2 variants among arriving international travelers.

**Methods:**

Between November 29, 2021-April 24, 2022, we enrolled arriving air travelers (≥18 years) from flights originating in 16 countries on 5 continents. At four airports, participants self-collected nasal swab samples that were pooled with 5–25 other samples by country of flight. Participants were also given a take-home saliva collection kit; saliva was collected 3-5 days after arrival and mailed back to the laboratory. SARS-CoV-2 reverse transcription–polymerase chain reaction (RT-PCR) was performed on all samples at the laboratory. Positives underwent whole genome sequencing. Demographic, clinical, and travel information was collected.

**Results:**

We enrolled 28,656 travelers; median age was 42 years (interquartile range 31-55), 48% were female, and 99.4% self-reported COVID-19 vaccination. Overall, 19% (504/2,666) of pooled and 7.5% (285/3804) of individual samples were positive for SARS-CoV-2. Highest pool positivity of 46% occurred during January 3–10, 2022 (Figure 1). Omicron variant accounted for 97% of sequences (Figure 2). We detected the earliest reporting of Omicron sub-lineages BA.2 and BA.3 (7 and 43 days earlier than reported elsewhere) in the United States and North America, respectively. During April 4–18, we detected an increasing trend of pool positivity among travelers on South African flights, detecting one of the first US-reported BA.4 sub-lineages consistent with early surge of cases in South Africa. Weekly pooled positivity for travelers on South African flights aligned with World Health Organization (WHO)-reported 7-day COVID-19 incidence rates over the same period (Figure 3).

**Conclusion:**

This genomic sequencing surveillance platform is a model for traveler-based SARS-CoV-2 genomic surveillance that can be used as an early warning system to detect future outbreaks and pandemics.

**Disclosures:**

**Renee Wegrzyn, PhD**, Ginkgo Bioworks: Stocks/Bonds **Robert C. Morfino, MBA**, Ginkgo Bioworks Inc: Employee|Ginkgo Bioworks Inc: Stocks/Bonds **Scott Milford, n/a**, XpresSpa Group, Inc: Stocks/Bonds **Scott Milford, n/a**, XpresSpa Group, Inc: Stocks/Bonds **Ezra T. Ernst, n/a**, XpresSpa Group, Inc: Ownership Interest|XpresSpa Group, Inc: Stocks/Bonds **William W. Darrow, n/a**, XpresSpa Group, Inc: Stocks/Bonds **Siyao Lisa Li, n/a**, Ginkgo Bioworks, Inc: Grant/Research Support|Ginkgo Bioworks, Inc: Stocks/Bonds **Thomas Aichele, n/a**, Ginkgo Bioworks, Inc: Grant/Research Support|Ginkgo Bioworks, Inc: Stocks/Bonds **Andrew Rothstein, n/a**, Ginkgo Bioworks, Inc: Grant/Research Support|Ginkgo Bioworks, Inc: Stocks/Bonds **Benjamin Rome, MBA**, Ginkgo Bioworks, Inc: Grant/Research Support|Ginkgo Bioworks, Inc: Stocks/Bonds **Gabrielle Woronoff, PhD**, Ginkgo Bioworks, Inc: Grant/Research Support|Ginkgo Bioworks, Inc: Stocks/Bonds **Keith Robison, PhD**, Ginkgo Bioworks, Inc: Grant/Research Support|Ginkgo Bioworks, Inc: Stocks/Bonds **Dongjuan Dai, PhD**, Ginkgo Bioworks, Inc: Grant/Research Support|Ginkgo Bioworks, Inc: Stocks/Bonds **Allison Hicks, PhD**, Ginkgo Bioworks: I am a current employee|Ginkgo Bioworks: Stocks/Bonds **Bryan Cosca, n/a**, Ginkgo Bioworks, Inc: Grant/Research Support|Ginkgo Bioworks, Inc: Stocks/Bonds **Alex Plocik, PhD**, Ginkgo Bioworks, Inc: Grant/Research Support|Ginkgo Bioworks, Inc: Stocks/Bonds **Birgitte Simen, PhD**, Ginkgo Bioworks: I am a current employee|Ginkgo Bioworks: Stocks/Bonds.

**Figure 1.**
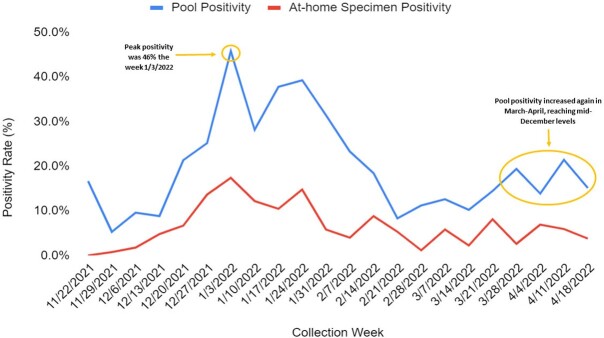
Weekly positivity of pooled and individual specimens obtained through traveler-based genomic surveillance for SARS-CoV-2, November 28, 2021-April 18, 2022

**Figure 2.**
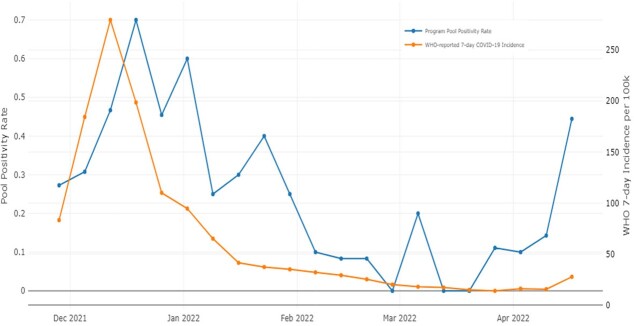
Comparison of weekly pooled test positivity in travelers arriving in the United States from South Africa and COVID-19 incidence rates in South Africa reported by the World Health Organization (WHO) –November 29, 2021-April 24, 2022

**Figure 3.**
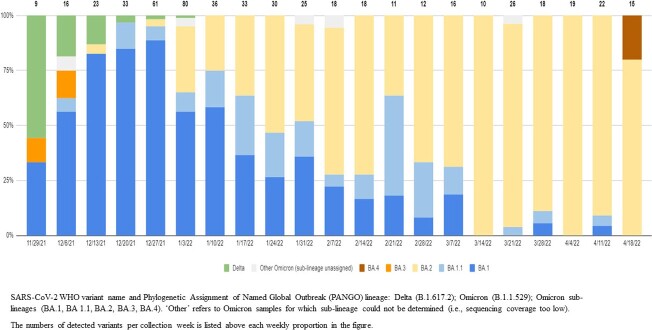
Proportions of SARS-CoV-2 variants detected through traveler-based genomic surveillance for SARS-CoV-2, Week of November 29, 2021-April 18, 2022

